# Subchorionic hematoma and pregnancy outcomes in patients with threatened miscarriage

**DOI:** 10.12669/pjms.38.3.4283

**Published:** 2022

**Authors:** Sumaira Naz, Sheikh Irfan, Tahira Naru, Ayesha Malik

**Affiliations:** 1 Dr. Sumaira Naz, FCPS. Department of Obstetrics & Gynecology, Aga Khan University Hospital, Karachi, Pakistan; 2 Dr. Sheikh Irfan, MPH. Department of Obstetrics & Gynecology, Aga Khan University Hospital, Karachi, Pakistan; 3 Dr. Tahira Naru, FAOG, MD. Department of Obstetrics & Gynecology, Aga Khan University Hospital, Karachi, Pakistan; 4 Dr. Ayesha Malik, MRCOG. Department of Obstetrics & Gynecology, Aga Khan University Hospital, Karachi, Pakistan

**Keywords:** Threatened Miscarriage, Subchorionic Hematoma, Small for gestational age, Preeclampsia

## Abstract

**Objective:**

To compare maternal and perinatal outcomes in patients with threatened miscarriage with or without subchorionic hematoma (SCH) at a tertiary care hospital.

**Methods:**

This retrospective cohort study was conducted at Aga Khan University Hospital. The study included 200 patients of <20 weeks singleton pregnancy with threatened miscarriage from January 2016 till December 2018. These patients were divided into two groups based on the presence (study group) or absence of subchorionic hematoma (control) on ultrasound imaging. Baseline demographic data, and obstetric outcomes were compared for the two groups.

**Results:**

The incidence of subchorionic hematoma was observed to be 30.5% (61/200). Most of the patients of SCH and non SCH groups presented in first trimester. Age and BMI were similar for both groups however there were more multigravida patients in the SCH group (63%versus 46.7%, P=0.12). A higher number of patients in the SCH group ended up in spontaneous miscarriage in contrast to patients with no SCH (13%versus6.1%, P=0.07) and also had a greater proportion of small for gestational age (SGA) babies (8.9%versus3.9%) though no statistical significance was observed. There were more preeclamptic patients in SCH group as compared to non SCH group (4.8%versus0.7%) and the trend was statistically significant(P=0.05). However, no significant correlation of hematoma size and adverse pregnancy outcomes was found in SCH group.

**Conclusion:**

Our study shows that women with threatened miscarriage having SCH are at a higher risk of having preeclampsia and SGA and hence these pregnancies warrant greater surveillance.

## INTRODUCTION

Threatened miscarriage is the most common complication in the first half of pregnancy affecting 20-25% of women.^1^ Diagnosis of threatened miscarriage is established when there is a viable pregnancy with vaginal bleeding and a closed cervix.^2^

Any woman who presents with threatened miscarriage, ultrasound imaging is performed to ascertain the viability, location of the placenta and the presence or absence of sub chorionic hematoma. Subchorionic hematoma (SCH) is defined as a sonographically-detected, collection of blood between the chorion and the endometrium. Although, SCH may happen spontaneously, the exact aetiology is uncertain. They are believed to result from partial detachment of the chorionic membranes from the uterine wall.^3^ The other possible explanation could be affected trophoblast invasion and impaired change in spiral arteries at the time of implantation. The incidence of hematoma detected on ultrasound varies between 0.46-39.5%.^4^ Almost 20% of women that present with threatened miscarriage have a sub chorionic hematoma.^5^

Threatened miscarriage has been linked with increased risk of hypertensive disorders of pregnancy, preterm pre labor rupture of membranes(PPROM), spontaneous preterm labor, antepartum hemorrhage (APH), intra uterine growth retardation and Caesarean Section.^2^,^6^ Fetal loss is also described in about one quarter of threatened miscarriage cases especially from low socioeconomic background.^7^ Some studies have mentioned SCH as a causal factor for adverse outcomes whereas other studies observed no association between SCH and affected maternal and perinatal outcomes.^4^,^8^,^9^ One meta-analysis demonstrated an increased risk of early and late pregnancy loss, placental abruption, and PPROM in patients with SCH.^10^ However, Li Q et al.^11^ in a recent meta-analysis identified increased risk of miscarriage with SCH but found no effect on premature delivery rate and delivery mode. Similarly, controversies exist regarding the correlation of SCH volume, and the simultaneous presence of vaginal bleeding with adverse obstetric outcomes.^8^ Pedersen and Mantoni^12^ stated that presence of large hematomas does not affect pregnancy outcomes whereas Bennett et al.^13^ highlighted that factors like maternal age, size of the hematoma, and gestational age affects fetal outcome.

These conflicting results emphasized the need to consider whether it’s the occurrence of first trimester bleed or the presence of subchorionic hematoma that augments the risk for adverse outcomes in threatened miscarriage. Furthermore, local data is deficient to evaluate significance of SCH in these patients. In our setting, a number of pregnant patients present with vaginal bleeding during early pregnancy and diagnosed to have threatened miscarriage. This study will help to comprehend any added risks to patients with SCH and threatened miscarriage and provide an insight to the care givers to counsel these patients for anticipated adverse outcomes.

## METHODS

This retrospective cohort study was conducted in Obstetrics& Gynecology department at Aga Khan University Hospital, Karachi, Pakistan. After attaining exemption from ethical review board (ERC# 2019-1837-4975, September 5^th^, 2019), medical records of all patients were reviewed who were diagnosed with threatened miscarriage (n=286) during first trimester or early second trimester and admitted from January 2016 to December 2018. Data was retrieved from hospital inpatient database using coding for threatened miscarriage and subchorionic hematoma. The inclusion criteria were singleton alive intrauterine pregnancy with gestational age <20 weeks and bleeding per vaginum (threatened miscarriage) with or without SCH. Patients were excluded if having diagnosis of miscarriage other than threatened miscarriage, absent fetal cardiac activity, gestational age ≥20 weeks, multiple pregnancy, known hypertensive status, any bleeding disorders, cases of detected congenital anomalies, prior history of recurrent miscarriages and preterm labor. Those who lost to follow up for delivery were also excluded from the selected patients.

There were 200 patients who met inclusion criteria. These patients were then divided into two groups based on the presence (study group) or absence of subchorionic hematoma (control group) on ultrasound imaging of pelvis. The patients who had threatened miscarriage without sub chorionic hematoma were recruited as control group(n=139) and the patients with SCH (n=61) were included in study group. A structured proforma was used to gather data regarding demographics, pregnancy course, ultrasound information for SCH and maternal and perinatal outcomes. Both groups were compared for pregnancy outcomes including miscarriage, APH including abruptio placentae& placenta previa, PIH, preeclampsia, intrauterine fetal demise, preterm labor, PPROM and mode of delivery. In patients whose pregnancies continued and resulted in delivery, term or preterm status, birth weight, Apgar score, NICU admission and neonatal death were also compared between the two groups. SCH patients were also evaluated for correlation of hematoma size (<5cm or >5cm) with adverse pregnancy outcomes.

### Statistical Analysis

All the data was entered and analyzed in SPSS version 19.0. Categorical variables in the statistical analysis were assessed by chi square analysis or two-tailed Fisher exact test in cases of small– expected cell frequencies. Differences in continuous variables were evaluated by a two-tailed Student t test. P-values less than .05 was considered statistically significant and the 95% confidence intervals was calculated.

## RESULTS

In this study, a total of 200 pregnant patients with threatened miscarriage were recruited. Out of these, sixty-one patients had sonographic evidence of SCH while 139 patients were without hematoma. The incidence of SCH was found to be 30.5% (61/200) and in 78% patients’ size of hematoma was less than 5cm whereas 21% had hematoma more than 5cm.

Demographic features of participants with and without SCH are shown in Table-I. The frequency of different age group intervals was similar for patients of both groups. In SCH group, 39% of patients were aged between 25 to 29 years versus 41% patients in non SCH group. Similarly, in SCH group 16% of all patients were above 35 years of age while 15% were 35 years of age. There was more multigravida in the SCH group (63%) compared to the non-hematoma group (46.7%). However, this difference did not reach statistical significance (p=0.12). Most of the patients presented in first trimester as compared to second trimester. This trend was similar both for SCH (67% versus 32%) and non SCH group (64% versus 36%). In both the groups, the frequency of overweight and obese women was similar.

**Table-I:** Clinical characteristics of study participants among study and control groups (n=200).

Demographics	* TM without SCH (Control Group)	TM with SCH (Study Group)	P Value* (p<0.05)
***Age in years***			
18-24	26 (18.7%)	11 (18%)	0.991
25-29	57 (41%)	24 (39%)	
30-34	35 (25%)	16 (26%)	
>35	21 (15%)	10 (16.3%)	
***Parity***			
Primigravida	74 (53.2%)	22 (36%)	0.012
Multigravida	65 (46.7%)	39(63%)	
***Gestational Age***			
First trimester (1-13 weeks)	89 (64%)	41 (67.2%)	0.748
Second trimester (14 -26weeks)	50 (35.9%)	20 (32.7%)	
***BMI***			
<18	6 (4.3%)	2 (3%)	0.757
18-24.9	42 (31%)	23 (37.7%)	
25-29.9	58 (41%)	20 (32%)	
30-35	28 (20.1%)	14 (22.9%)	
>35	5 (3 %)	2 (3.2%)	

* TM: Threatened Miscarriage SCH: Subchorionic Hematoma P Value: significant if < 0.05.

About 13% patients in SCH group ended up having a miscarriage. Table-II While in comparison, the non SCH group had 6.1 % of index pregnancies ending in miscarriage. There was a trend towards statistical significance (0.07). The frequency of preterm labor was broadly comparable in both the groups with a non-significant p-value of 0.4. In SCH group, 82% of participants presented with preterm labor compared to 76% patients in the other group. A similar comparable trend was seen for PPROM among both the group with p value of 0.31. In each group one patient had APH due to abruption.

**Table-II:** Maternal outcomes in between study and control groups (n=200).

Obstetric Outcome	TM without SCH (Control Group)	TM with SCH (Study Group)	P value (P <0.05)
***Miscarriage***			
Yes	8(6.1%)	8 (13%)	0.07
No	131(93.8%)	53(86.8%)	
***Preterm Labor***			
No	106 (76.2%)	50 (81.96 %)	0.459
Yes	33 (23.7%)	11 (18%)	
***PPROM***			
No	122 (87.7%)	57 (93.4%)	0.318
Yes	17 (12.2%)	4 (6.5%)	
***Antepartum Hemorrhage***			
No	138 (99%)	60 (98.3 %)	0.518
Yes	1 (0.7 %)	1 (1.64%)	
***SGA***			
No	121 (96%)	42 (91.3%)	0.251
Yes	5 (3.9%)	4 (8.9%)	
***Preeclampsia***			
No	138(99.2%)	58(95%)	0.05
Yes	1 (0.7%)	3 (4.8%)	
***Mode of Delivery***			
SVD	51 (40.8%)	23 (51%)	0.151
LSCS	66 (52.8%)	22 (48.8%)	
Assisted Vaginal Delivery	08 (6.4%)	0	

There were smaller for gestational age (SGA) fetuses in the SCH group (8.9%) compared to 3.9% in the non-hematoma group. Though this could not reach statistical significance due to small numbers in each cell. The number of preeclamptic patients in the hematoma group was higher (4.8%) compared to the other group (0.7%) and the trend was statistically significant(p=0.05). The frequency of cesarean section was similar in both the groups; with 48.8% of patients in the SCH group ending up in lower segment cesarean section (LSCS) while the frequency of operative delivery was 52.8% in the non SCH group (Table-II).

Neonatal outcomes of the two groups are shown in Table-III. The frequency of normal birth weight babies was similar for both the groups. The frequency of low birth weight in the SCH and non SCH was 15.6% and 21.6% respectively. There was no difference observed in the 5-minute APGAR score of neonates for both the groups. However,11% of neonates from the SCH group needed NICU admission versus 8% of newborns in the non SCH group.

**Table-III:** Neonatal outcomes in between study and control groups (n=200).

Neonatal Outcome	TM without SCH (Control Group)	T M with SCH (Study Group)	P value (P value <0.05)
***Birth Wt. (Kg)***			
<2.5	27 (21.6%)	07 (15.5%)	0.136
2.5-3.5	95 (76%)	34 (75.5%)	
>3.5	3 (2.4%)	4 (8.8%)	
***Apgar Scores***			
3.00	1 (0.8 %)	0	0.674
7.00	0	3 (6%)	
8.00	2 (1.63%)	1 (2%)	
9.00	119 (97.5 %)	44 (91.6 %)	
***NICU Admission***			
No	114 (91.0%)	40 (88.8%)	0.548
Yes	10 (8 %)	05 (11.1%)	

## DISCUSSION

Our study results showed SCH in 30% of patients with threatened miscarriage and this was comparable to 43% reported by Dongol A et al.^14^ The salient findings of our study were that there were more multigravida patients in the SCH group. In addition, a higher number of patients in the hematoma group ended up in spontaneous miscarriage in contrast to patients with no SCH and also had a greater proportion of SGA babies and pre-eclampsia compared to the group with only threatened miscarriage.

Study by Peixoto et al.^15^, also found parity to be higher with sub chorionic hematoma. Similarly, a polish study^16^ also found a higher proportion of multipara women in the hematoma group. This may be due to the multigravida group having a greater proportion of women with advance maternal age, which is also a risk factor for SCH.^17^

Our results showed a greater proportion of women in the hematoma group to eventually have spontaneous miscarriage compared to the non-hematoma group. This finding is consistent with previous studies. Tuuli et al.^10^ pooled the results of five studies and concluded that women with SCH have a two-fold increase risk of spontaneous miscarriage in contrast to women with only threatened miscarriage. Similarly, a local study highlighted strong association of SCH with miscarriage (37.9% vs. 9.7%, P-value <0.05) as compared to non SCH group.^18^ Our findings support the postulated hypothesis that the mechanical effect of SCH can cause miscarriage by causing detachment of the sac from the endometrium.^6^

Our study showed that greater proportion of women in the hematoma group had SGA fetuses compared to their non-hematoma counterparts whereas, previous studies regarding SGA as an outcome have conflicting results. Our findings are consistent with a study by Ozkaya et al.^19^, that described an increased risk of SGA in the hematoma group. Similarly, a study done by Nagy et al. which had 187 patients with SCH, and 6488 controls showed SGA was significantly higher in the SCH group (7% versus 3% [ p=0.002]).^20^ In contrast, a Russian study by Bushtyreva et al., which included 115 women with SCH, and 79 controls did not find any significant association between SGA and SCH.^9^

Our study did not show any statistical increase in the number of preterm deliveries in the SCH group. Existing literature shows conflicting evidence with regards to association of SCH and preterm delivery. A study done by Peixoto AB et al.^15^, demonstrated an equal rate of 16% preterm deliveries for both the SCH and non SCH group. Similarly, a study conducted by Irina O Bushtyreva did not find any correlation between SCH diagnosed in first trimester and increase rate of preterm delivery.^9^ However, there also exists contrary evidence. A retrospective cohort study conducted by Normal et al.^21^ showed an increase risk of preterm delivery less than 34 weeks in patients diagnosed with subchorionic hematoma before 22 weeks: 4.2% compared with 2.7%, (OR 1.7, 95% CI 1.3–2.4, P.01).

A significant association between preeclampsia and SCH was observed in our study. This is consistent with the outcomes reported by Nagy et al.^20^ that women with SCH were at a fourfold higher risk of preeclampsia compared to women in the control group (RR 4.0,95% CI (2.4, 6.7)). A local study has also described similar association.^18^ However, our results do contradict with few studies. The retrospective cohort study done by Araujo et al.^15^, showed no association between subchorionic hematoma and hypertension in pregnancy. The study done by Hashem et al.^22^ in India did show an association between SCH and SGA but not with preeclampsia. Our study has shown an increase frequency of both SGA and preeclampsia in the SCH group compared to the control group.^10^ To explain these findings, it is important to discuss the underlying mechanism.

Though the phenomenon is incompletely understood, one possible explanation is the occurrence of premature perfusion of the intervillous space due to the presence of SCH prior to the completion of placental adaptation to cope with oxidative stress.^10^,^23^ Other possible explanations could be shallow trophoblastic invasion with inadequate angiogenesis causing friable blood vessels. These fragile blood vessels predispose to subchorionic hemorrhage.^11^,^23^ Furthermore, we found no significant correlation of hematoma size with adverse pregnancy outcomes. This is consistent with the findings reported by Naqvi M et al.^24^

### Strength of our study

The strength of our study is that to our knowledge there are limited studies from South Asia addressing this issue and only one study from Pakistan has evaluated association of SCH with adverse pregnancy outcomes.^18^ Other local studies have addressed management^25^ and outcomes of threatened miscarriage^6^ without SCH or focused on the sonographic features of SCH.

### Limitations of our study

It includes its retrospective nature and relatively small sample size. The small study sample resulted in showing results that were not statistically significant though they were clinically important.

A number of studies have postulated defective placentation as underlying mechanisms of SCH.^2^ It is interesting to note that we also have found an increase frequency of placental mediated diseases in this group, that is, Preeclampsia and SGA. This opens up the opportunity for screening for Preeclampsia in first trimester using Uterine artery Dopplers and possible use of Aspirin later if the per vaginal bleeding has settled.

## CONCLUSION

Our study shows that women with threatened miscarriage having SCH are at a higher risk of having preeclampsia and SGA and hence these pregnancies warrant greater surveillance.

### Author`s Contribution:

**SNAZ** conceived, drafting, data collection, manuscript writing, responsible for data integrity and authenticity.

**ISH** did statistical analysis and review of manuscript.

**TNAR** reviewed and final approval of manuscript.

**AM** contributed to designing, results analysis, manuscript writing and editing.
